# Newly diagnosed cardiovascular disease in patients treated with immune checkpoint inhibitors: a retrospective analysis of patients at an academic tertiary care center

**DOI:** 10.1186/s40959-021-00097-9

**Published:** 2021-03-18

**Authors:** Nida Waheed, Michael G. Fradley, David L. DeRemer, Ahmad Mahmoud, Chintan P. Shah, Taimour Y. Langaee, Gloria P. Lipori, Keith March, Carl J. Pepine, Rhonda M. Cooper-DeHoff, Yonghui Wu, Yan Gong

**Affiliations:** 1 Division of Cardiology, Department of Medicine, Emory University School of Medicine, Atlanta, GA USA; 2 Cardio-Oncology Center of Excellence, Division of Cardiology, Department of Medicine, Perelman School of Medicine at the University of Pennsylvania, Philadelphia, PA USA; 3 Department of Pharmacotherapy and Translational Research, College of Pharmacy, University of Florida, PO Box 100486, 1345 Center Drive, Gainesville, FL 32610-0486 USA; 4 UF Health Cancer Center, Gainesville, Florida USA; 5 Division of Cardiovascular Medicine, Department of Medicine, University of Florida, Gainesville, Florida USA; 6 Division of Hematology and Oncology, Department of Medicine, University of Florida, Gainesville, Florida USA; 7 Center for Pharmacogenomics and Precision Medicine, College of Pharmacy, University of Florida, Gainesville, Florida USA; 8 UF Health, Gainesville, Florida USA; 9 Department of Pharmaceutical Outcomes and Policy, College of Pharmacy, University of Florida, Gainesville, Florida USA; 10 Health Outcome and Biomedical Informatics, College of Medicine, University of Florida, Gainesville, Florida USA

**Keywords:** Cardio-oncology, Immune checkpoint inhibitors, Cardiomyopathy, Heart failure

## Abstract

**Background:**

Immune checkpoint inhibitors (ICIs) are a novel class of anticancer agents that have demonstrated clinical response for both solid and hematological malignancies. ICIs are associated with development of immune-related adverse events including cardiotoxicity. We estimated the incidence of newly diagnosed cardiovascular disease in patients treated with ICIs at a large, tertiary care center.

**Methods:**

All patients with a cancer diagnosis who received any ICI treatment in the University of Florida’s Integrated Data Repository from 2011 to 2017 were included. Cardiovascular disease was defined as a new ICD diagnosis code for cardiomyopathy, heart failure, arrhythmia, heart block, pericardial disease, or myocarditis after initiation of ICI treatment.

**Results:**

Of 102,701 patients with a diagnosis of malignancy, 424 patients received at least one ICI. Sixty-two (14.6%) patients were diagnosed with at least one new cardiovascular disease after initiation of ICI therapy. Of the 374 patients receiving one ICI, 21 (5.6%) developed heart failure. Of the 49 patients who received two ICIs sequentially, three (6.1%) developed heart failure and/or cardiomyopathy. Incident cardiovascular disease was diagnosed at a median of 63 days after initial ICI exposure. One patient developed myocarditis 28 days after receiving nivolumab. Mortality in ICI treated patients with a concomitant diagnosis of incident cardiovascular disease was higher compared to those who did not (66.1% vs. 41.4%, odds ratio = 2.77, 1.55–4.95, *p* = 0.0006).

**Conclusions:**

This study suggests a high incidence of newly diagnosed cardiovascular disease after the initiation of ICI therapy in a real-world clinical setting.

**Supplementary Information:**

The online version contains supplementary material available at 10.1186/s40959-021-00097-9.

## Background

Immune checkpoint inhibitors (ICIs) have revolutionized the management of a diverse spectrum of solid and hematological malignancies previously associated with poor prognosis. Immune checkpoint blockade removes inhibitory signals of T-cell activation enabling tumor-reactive T cells to mount an effective antitumor response by overcoming regulatory mechanisms [1]. Currently, FDA-approved ICIs are inhibitors of either the cytotoxic T-cell lymphocyte-associated protein-4 (CTLA-4) or the programmed cell death receptor 1 (PD-1) or its ligand (PD-L1). Robust research efforts evaluating other checkpoint targets such as lymphocyte-activation gene-3 (LAG-3) [2] and T-cell immunoglobulin and mucin-domain containing-3 (TIM-3) [3] are ongoing.

ICIs have been reported to cause a range of immune-related adverse events (irAE), mostly involving the skin, endocrine system, liver, lungs, and gastrointestinal tract. These targeted therapies affect specific signaling pathways that can also induce cardiotoxicity. IrAEs occur due to inhibition of immune checkpoints that boost physiological barriers against autoimmunity, leading to local and systemic autoimmune responses [4]. Fulminant myocarditis is currently the most recognized irAE, but complete heart block, conduction abnormalities, pericarditis, stress-induced cardiomyopathy, and left ventricular dysfunction have also been reported [5–7]. Limited data are available on the incidence of new cardiovascular disease (CVD) after ICI initiation, and there is scarce evidence to guide prevention, surveillance, and treatment [8–10]. The reported incidence of ICI-related myocarditis ranges from 0.06 to 1.14% of patients receiving ICIs [11, 12]. However, absence of systematic monitoring and coding mechanisms for cardiac events in immunotherapy trials suggest that cardiac irAEs may be under-reported [9]. Accordingly, we estimated the incidence of new CVD among patients treated with ICIs using electronic health records (EHRs) at a large tertiary care center.

## Methods

This was an observational cohort study using data extracted from EHRs. Supported by the University of Florida (UF) Clinical and Translational Science Institute (CTSI), the UF Health Integrated Data Repository (IDR) is a large-scale database that collects and organizes information from across the UF-Health clinical and research enterprises, thereby including most inpatient and outpatient care services. The IDR provides access to Health Insurance Portability and Accountability Act (HIPAA) compliant and Institutional Review Board (IRB) approved limited datasets that include demographics, medications, lab results, diagnosis, and clinical encounters. For this study, the UF IDR was queried to extract information relevant to all patients receiving anticancer drugs from 2011 to 2017. All patients with the International Classification of Disease, ninth and tenth revisions, clinical modification (ICD-9-CM 140–239.99) and (ICD-10-CM C00-D49) codes for malignancy were included.

The current study consisted of patients who had received at least one dose of any ICI including PD-1 inhibitors (pembrolizumab, nivolumab), PD-L1 inhibitors (atezolizumab, durvalumab), and CTLA-4 inhibitors (ipilimumab, tremelimumab). Baseline demographic information was collected at the encounter of the first ICI administration. Comorbidities such as history of hypertension, hyperlipidemia, diabetes, and ischemic heart disease were defined based on the presence of International Classification of Diseases, 9th revision, Clinical Modification (ICD-9-CM) and ICD-10-CM diagnosis codes prior to the first ICI prescription date.

New CVD was defined by ICD-9-CM and ICD-10-CM codes for cardiomyopathy, heart failure, myocarditis, arrhythmia, pericardial disease and heart block (Supplemental Table) entered by a clinician after the initiation of the ICI without prior history of incident cardiovascular condition. The performances of these computable phenotypes were previously reported in similar EHRs. The ranges of the estimates were 79–95% for sensitivity, 90–98.9% for specificity, 70–94% for positive predictive value, and of 95–99.4% for negative predictive value, respectively [13, 14]. Patients with existing diagnosis codes for cardiomyopathy, heart failure, myocarditis, arrhythmia, pericardial disease, and/or heart block before ICI initiation were considered to have pre-existing disease and were excluded from analysis.

Statistical analysis: Demographic and medical history information of those with and without a diagnosis of post-ICI CVD were compared using Student’s unpaired t-test for continuous variables and chi-square test for categorical variables as appropriate. The percentages of patients with CVD following exposure to each drug were estimated. Multivariable logistic regression analysis was performed to estimate the odds ratios (ORs) and 95% confidence intervals (CIs) for mortality adjusting for demographics, comorbidities, and ICIs. Covariates with univariate *p*-value of < 0.2 were considered in the multivariable logistic regression and variables with *p* < 0.05 were retained in the model. All analyses were performed in SAS v. 9.4 (Cary, NC). This study was approved by the University of Florida Institutional Review Board (IRB) (IRB# 201702876).

## Results

Of 102,701 patients with a diagnosis of malignancy, 424 patients received at least one ICI and their pertinent demographic and clinical characteristics are summarized in Table 1. Overall, the median age was 63 years, the majority were men (63.4%), 85.6% were non-Hispanic whites and 7.6% were non-Hispanic blacks. The most frequent cancer diagnoses were lung cancer (29.7%), melanoma (17.0%), and kidney cancer (12.7%) (Table 1). Almost half of the patients (49.5%) had hypertension, 30.2% had hyperlipidemia, 17.9% had diabetes, and 12.7% had ischemic heart disease before the initiation of ICI treatment as determined by ICD diagnosis codes. Exposures to other cancer medications are also summarized in Table 1.

**Table 1:** Demographic and clinical characteristics of patients treated with immune checkpoint inhibitors

Characteristics	All (***n*** = 424)	Newly diagnosed CVD (***n*** = 62)	No CVD (***n*** = 362)	P
Age (year)	62 ± 13.1	64.3 ± 10.2	62.1 ± 13.5	0.135
Sex				0.924
Women	155 (36.6%)	23 (37.1%)	132 (36.5%)	
Men	269 (63.4%)	39 (62.9%)	230 (63.5%)	
Race/Ethnicity				0.772
White (non-Hispanic)	363 (85.6%)	55 (88.7%)	308 (85.0%)	
Black (non-Hispanic)	32 (7.6%)	4 (6.5%)	28 (7.8%)	
Hispanic	16 (3.8%)	1 (1.6%)	15 (4.2%)	
Other	13 (3.1%)	2 (3.2%)	11 (3.1%)	
Primary cancer diagnosis				0.163
Lung cancer	126 (29.7%)	20 (32.3%)	106 (29.3%)	
Melanoma	72 (17.0%)	16 (25.8%)	56 (15.5%)	
Kidney cancer	54 (12.7%)	7 (11.3%)	47 (13.0%)	
Head and neck cancer	45 (10.6%)	6 (9.7%)	39 (10.8%)	
Urothelial carcinoma	34 (8.0%)	6 (9.7%)	28 (7.7%)	
Colorectal cancer	19 (4.5%)	4 (6.5%)	15 (4.1%)	
Gastrointestinal cancers (other)	8 (1.9)	1 (1.6%)	7 (1.9%)	
Hodgkin Lymphoma	7 (1.6%)	0	7 (1.9%)	
Other cancer	59 (13.9%)	2 (3.2%)	57 (15.8%)	
Cardiovascular risk factors				
Hypertension	210 (49.5%)	36 (58.1%)	174 (48.1%)	0.146
Ischemic heart disease	54 (12.7%)	9 (14.5%)	45 (12.4%)	0.649
Hyperlipidemia	128 (30.2%)	24 (38.7%)	104 (28.7%)	0.114
Diabetes	76 (17.9%)	14 (22.6%)	62 (17.1%)	0.301
Other cancer medications				
Doxorubicin	14 (3.3%)	3 (4.8%)	11 (3.0%)	0.464
Carboplatin	114 (26.9%)	18 (29.0%)	96 (26.5%)	0.68
Paclitaxel	88 (20.8%)	15 (24.2%)	73 (20.2%)	0.47
Cyclophosphamide	4 (0.9%)	1 (1.6%)	3 (0.8%)	0.555

Values were reported as mean ± standard deviation for continuous variables and frequency (%) for categorical variables

A single ICI treatment was prescribed to 374 patients. Of patients treated with PD-1 inhibitors, 217 (58%) received nivolumab and 123 (32.8%) were treated with pembrolizumab. Forty-nine patients received dual-agent therapy sequentially (39 with PD-1/PD-L1 inhibitor and CTLA-4 inhibitor combination); one patient received three different ICIs sequentially (Table 2).

**Table 2:** Newly diagnosed cardiovascular disease after ICI initiation

Immune Checkpoint Inhibitor	Cardiomyopathy, n (%)	Heart failure, n (%)	Arrhythmia, n (%)	Pericardial disease, n (%)	Heart block, n (%)	Myocarditis, n (%)	Any cardiotoxicity, n (%)	Median (IQR) time to cardiotoxicity, days
PD-1 inhibitors								
nivolumab (*n* = 217)	1 (0.46)	10 (4.61)	15 (6.91)	7 (3.23)	6 (2.76)	1 (0.46)	33 (15.21)	52 (37–203)
pembrolizumab (*n* = 123)	0	6 (4.88)	3 (2.44)	1 (0.81)	1 (0.81)	0	11 (8.94)	65 (30–175)
PD-L1 inhibitors								
atezolizumab (*n* = 17)	0	1 (5.88)	2 (11.76)	0	0	0	3 (17.65)	22 (2–172)
durvalumab (n = 4)	1 (25)	0	0	0	0	0	1 (25.00)	30
CTLA-4 inhibitor								
ipilimumab (*n* = 13)	0	4 (30.77)	1 (7.69)	0	1 (7.69)	0	6 (46.15)	709 (78–1469)
CTLA-4 + PD-1/PD-L1 inhibitor combination								
ipilimumab + nivolumab (*n* = 29)	2 (6.9)	1 (3.45)	4 (13.79)	0	1 (3.45)	0	7 (24.14)	95 (11–119)
ipilimumab + pembrolizumab (*n* = 7)	0	1 (14.29)	1 (14.29)	0	0	0	1 (14.29)	62
tremelimumab + durvalumab (n = 3)	0	0	0	0	0	0	0	
PD-1/PD-L1 dual sequential								
nivolumab - > pembrolizumab (n = 4)	0	0	0	0	0	0	0	
nivolumab - > atezolizumab (*n* = 3)	0	0	0	0	0	0	0	
pembrolizumab - > atezolizumab (n = 3)	0	0	0	0	0	0	0	
Three-drug sequential								
ipilimumab + nivolumab - > pembrolizumab (n = 1)	0	0	0	0	0	0	0	
Total (n = 424)	4 (0.94)	23 (5.42)	26 (6.13)	8 (1.89)	9 (2.12)	1 (0.24)	62 (14.62)	63 (30–175)

*IQR* inter-quartile range

Of the 424 ICI-treated patients, sixty-two (14.6%; 95% CI, 11.3–18.0) met the definition for new CVD after initiation of ICI therapy. There were no statistically significant differences between those with and without a new CVD in terms of demographics, cancer type, or comorbidities (Table 1). However, there was a noticeable trend of higher percentages of hypertension (58.1% vs. 48.1%, *p* = 0.146), hyperlipidemia (38.7% vs. 28.7%, *p* = 0.114) and diabetes (22.6% vs. 17.1%, *p* = 0.301) in the patients who developed new CVD compared to patients who did not develop CVD after ICI treatment. The most frequently diagnosed cardiac conditions were arrhythmia (*n* = 26, 6.1%) and heart failure (*n* = 23, 5.4%) (Table 2). Of the 374 patients receiving only one ICI, 21 (5.6%) developed heart failure and 21 (5.6%) developed arrhythmia. Overall, in the patients treated with a single ICI, the rates of new CVD were the highest in those treated with the CTLA-4 inhibitor ipilimumab (6/13 = 46.15%), compared to PD-L1 inhibitors (4/21 = 19.1%) and PD-1 inhibitors (44/340 = 12.94%) (*p* = 0.0031). Of the 39 patients receiving two ICIs sequentially (PD-1/PD-L1 inhibitor + CTLA-4 inhibitor), five patients (12.8, 95% CI, 2.3–23.3) developed cardiomyopathy, and two patients (5.1, 95% CI, 0.0–12.1) developed heart failure. Eight patients (1.9%) were diagnosed with pericardial disease, and seven patients (1.7%) were diagnosed with heart block (1st to 3rd degree) after PD-1 inhibitor therapy. Ten patients received PD-1/PD-L1 inhibitor therapy sequentially and one patient received three ICIs sequentially; none of these patients developed a new cardiovascular condition within the study period. New CVD was diagnosed at a median time of 63 days after initial ICI exposure (interquartile range: 30–175 days) (Table 2). One patient developed myocarditis at day 28 after receiving nivolumab.

Forty-three patients including 9 (14.5%) with and 34 (9.4%) without a new CVD diagnosis had both pre-ICI treatment and post-ICI treatment transthoracic echocardiograms. The baseline left ventricular ejection fraction (LVEF) were 58% and 56% in the two groups, respectively. Even through there was no significant difference in the mean changes in LVEF (*p* = 0.37) in those patients with new CVD (− 6.3%) compared to those without (− 0.8%), there was a trend that patients who developed CVD had more reduction in LVEF.

Of the 424 ICI-treated patients, 191 (45.1%) died during the study period. The median time from ICI initiation to death was 128 days with interquartile range of 66–277 days. History of ischemic heart disease (OR: 2.11, 1.14–3.89, *p* = 0.017), prior use of doxorubicin (OR: 4.86, 1.31–18.11, *p* = 0.0184) and carboplatin use (OR: 1.86, 1.19–2.92, *p* = 0.0068) were also associated with higher mortality. After adjusting for the history of ischemic heart disease, and prior use of doxorubicin and carboplatin, mortality in those who developed new CVD remained higher compared to those who did not (66.1% vs. 41.4%, adjusted OR: 2.77 and 95% CI: 1.55–4.95, *p* = 0.0006). There was no evidence that the mortality was lower in patients treated with cardioprotective agents such as beta-blockers, angiotensin converting enzyme (ACE) inhibitors, angiotensin receptor blockers (ARBs), and statins (Table 3).

**Table 3:** Use of cardioprotective agents and mortality

Drug class	Total (***n*** = 424)	Mortality (%)	p-value
Beta-Blockers	Yes (*n* = 252)	126 (50%)	0.013
	No (*n* = 172)	65 (37.8%)	
ACE Inhibitors	Yes (*n* = 146)	69 (47.3%)	0.507
	No (*n* = 278)	122 (43.9%)	
Angiotensin Receptor Blockers	Yes (*n* = 55)	26 (47.3%)	0.722
	No (*n* = 369)	165 (44.7%)	
Statins	Yes (*n* = 164)	75 (45.7%)	0.822
	No (*n* = 260)	116 (44.6%)	

*ACE* angiotensin converting enzyme

## Discussion

In this study, we observed that approximately 15% of patients receiving ICI therapy developed new CVD. The most commonly observed CVD were heart failure and arrhythmia. As suggested in previous reports [15, 16], the incidence of myocarditis was very low: only one patient (0.24%) developed myocarditis. The time to myocarditis for this patient was 28 days after initiation of nivolumab, which was consistent with the reported median time to onset of 30 days by Salem and colleagues [15]. This relatively low prevalence may be related to inadequate screening, particularly since the study includes data starting from 2011, when the autoimmune side effects of ICIs were just being recognized in a clinical setting. However, the observed incidence of other manifestations of CVD was higher than previously suggested [15]. Interestingly, a recent study [17] reported that compared to non ICI treated cancer control patients, cancer patients treated with ICI had increased incidence of myocardial infarction, ischemic stroke, coronary revascularization, which is consistent with our finding. This study also demonstrated the ICIs may lead to accelerated atherosclerosis which may help to explain some of the incident CVD seen in our study [17].

Estimates of the incidence of ICI-induced cardiotoxicity vary substantially across reports. This might be explained, in part, by variations in case definitions and a specific focus on certain cardiac syndromes (e.g., myocarditis). Other case series on ICI-induced cardiotoxicity suggest that cardiomyopathy, myocarditis, and conduction abnormalities are under-reported [18]. The manufacturer of both ipilimumab and nivolumab reported myocarditis (0.09%) from detailed clinical trial safety data but other cardiovascular irAEs were later described in case reports [19–22].

Several studies have also characterized cardiac irAEs and their incidence. Myocarditis was one of the first recognized ICI-related AEs and has been the most studied of the ICI-related cardiotoxicities [16]. A multicenter registry including patients from the US, Canada, and Germany and found that the prevalence of myocarditis after ICI therapy was 1.14% with a median time of onset of 34 days, whereas another study reported a median time of 65 days from initiation of treatment [12, 23]. Pooled Food and Drug Administration (FDA) data on reported ICI-related adverse events in clinical trials suggested that the risks of cardiomyopathy, arrhythmia, myocarditis, and pericardial disease were 0.53, 5.56, 0.03, and 0.7%, respectively [24]. A meta-analysis of clinical trials of PD-1 inhibitors (nivolumab and pembrolizumab) and PD-L1 inhibitors (atezolizumab, avelumab and durvalumab) for treatment of non-small cell lung cancer also reported lower cardiovascular adverse event rates (1% for cardiorespiratory arrest, 2% for heart failure, 1% for myocardial infarction, and 2% for strokes) [25]. A case series of 30 patients with ICI-related cardiotoxicity, suggested the most frequently observed cardiotoxicities were reduced LVEF, arrhythmias, and pericardial disease with almost 80% of patients having left ventricular systolic dysfunction [23]. On the other hand, patients with ICI-related myocarditis have normal LVEF in 50% of the cases [12].

The incidence of irAEs has been noted to be dose-dependent after ipilimumab and pembrolizumab with greater toxicity at higher dose levels [9]. The differences in incidence of cardiac irAEs reported may be attributable to dose of ICI and future studies should provide details on ICI dosage, number of chemotherapy cycles, and their timing. Dosing of ICIs in clinical practice follows a predominantly fixed-dosing strategy (nivolumab – 240 mg, pembrolizumab – 200 mg) and extended dosing intervals (Q2 -Q3 weeks).

Several studies have suggested a potential role for the early initiation of cardioprotective medications including beta-blockers and angiotensin system inhibitors to prevent the development of cardiotoxicity associated with anthracyclines and trastuzumab [26–28]. Beta-blockers are not a consideration for ICI-related cardiotoxicities. The standard of care for ICI irAEs and for ICI-related myocarditis is high-dose corticosteroids [10]. Interestingly, in our study we found that baseline beta-blocker use was associated with increased mortality. There is no reason to consider beta-blockers themselves problematic in patients treated with ICI, rather they likely are a marker of a sicker population with more baseline CVD and/or risk factors.

There are several limitations to our study that should be noted. First, only one patient out of 62 with newly diagnosed CVD presented with myocarditis, the commonly recognized and more serious form of ICI cardiotoxicity. Second, due to the diversity of cancer patients treated with ICI, it was very difficult to select a group of cancer patients with similar cardiovascular comorbidity as controls. Also, this is a study of retrospectively collected clinical data from ICD codes. As such we were not able to confirm these findings with direct evaluation of the electronic medical records themselves. The use of ICD code groups such as “arrhythmia” and “heart failure” represent a heterogenous collection of diseases thereby impacting the interpretation of the findings. It is also important to recognize that while we excluded patients with baseline CVD, we cannot conclude from these data that new CVD is a direct result of the ICI exposure and a cardiotoxicity of therapy. Given the limited number of echocardiograms available, the lack of control group and the absence of a meaningful number of global strain studies, speculation regarding the role of diagnostic imaging in this patient population cannot be inferred from the present study. Another limitation is that there was no compliance assessment of medication use and the dose and duration of ICI use were not easily captured. We only had one patient diagnosed with ICI-related myocarditis, so we could not evaluate the effect of corticosteroids [29] or CTLA-4 agonist abatacept [30] on the outcome of myocarditis patients. Finally, while we identified increased mortality in those patients that developed CVD while on a ICI, we cannot assert this was directly related to the development of the CV disorder due to various competing risks of mortality in the population. Moreover, we are unable to establish the specific cause of death due to limitation of the dataset and this further impacts the interpretation of these findings. Finally, we also cannot rule out the possibility of selection and ascertainment biases. As such, due to these various limitations, the data presented should be considered hypothesis-generating only and not lead to definitive conclusions.

## Conclusions

This study suggests a high incidence of newly diagnosed CVD after the initiation of ICI therapy. The results of this analysis suggest that the incidence of ICI-associated cardiotoxicity may be higher than previously suggested. To better address this important knowledge gap, baseline cardiac assessment may be helpful for certain high-risk individuals (e.g., receiving combination ICI therapy, rapid decline in global longitudinal strains or a history of cardiac disease). Prospective studies are required to better characterize the incidence of specific cardiotoxicities and identify risk factors as well as long-term complications.

## Supplementary Information

**Additional file 1: Table S1.** ICD codes for newly diagnosed cardiovascular diseases after Immune Checkpoint Inhibitors treatment initiation.

## Data Availability

The data that support the findings of this study are available from OneFlorida but restrictions apply to the availability of these data, which were used under license for the current study, and so are not publicly available. Data are however available from the authors upon reasonable request and with permission of OneFlorida with appropriate IRB approval.

## Abbreviations

ICI: Immune checkpoint inhibitor
CTLA-4: Cytotoxic T-cell lymphocyte-associated protein-4
PD-1: The programmed death receptor 1
PD-L1: PD1 ligand
irAE: Immune-related adverse events
EHR: Electronic health records
UF: University of Florida
CTSI: Clinical and Translational Science Institute
IDR: Integrated Data Repository
HIPAA: Health Insurance Portability and Accountability Act
IRB: Institutional Review Board
ICD: International Classification of Disease
CM: Clinical Modification
OR: Odds ratio
CI: Confidence intervals
CVD: Cardiovascular disease
FDA: Food and Drug Administration
LVEF: Left ventricular ejection fraction
ACE: Angiotensin converting enzyme
ARB: Angiotensin receptor blocker
