# Supplementary material for: Newly diagnosed cardiovascular disease in patients treated with immune checkpoint inhibitors: a retrospective analysis of patients at an academic tertiary care center
Source: Cardiooncology. 2021 Mar 18;7:10. doi: 10.1186/s40959-021-00097-9 (PMC7977591; doi:10.1186/s40959-021-00097-9)
Supplement: Supplementary file 1 — Additional file 1: Table S1. ICD codes for newly diagnosed cardiovascular diseases after Immune Checkpoint Inhibitors treatment initiation. [file 40959_2021_97_MOESM1_ESM.docx]

**Supplemental Table: ICD codes for newly diagnosed cardiovascular diseases after Immune Checkpoint Inhibitors treatment initiation.**

| **Cardiotoxicity** | **ICD 9 codes** | **ICD 10 codes** |
| --- | --- | --- |
| Cardiomyopathy | 425.4 Other primary cardiomyopathies  425.8 Cardiomyopathy in other diseases classified elsewhere  425.9 Secondary cardiomyopathy, unspecified | [I42.0](http://www.icd10data.com/ICD10CM/Codes/I00-I99/I30-I52/I42-/I42.0) Dilated cardiomyopathy  [I42.5](http://www.icd10data.com/ICD10CM/Codes/I00-I99/I30-I52/I42-/I42.5) Other restrictive cardiomyopathy  [I42.7](http://www.icd10data.com/ICD10CM/Codes/I00-I99/I30-I52/I42-/I42.7) Cardiomyopathy due to drug and external agent  [I42.8](http://www.icd10data.com/ICD10CM/Codes/I00-I99/I30-I52/I42-/I42.8) Other cardiomyopathies  [I42.9](http://www.icd10data.com/ICD10CM/Codes/I00-I99/I30-I52/I42-/I42.9) Cardiomyopathy, unspecified |
| Heart Failure | 428 Heart failure  428.0 Congestive heart failure, unspecified  428.1 Left heart failure  428.2 Systolic heart failure  428.20 Systolic heart failure, unspecified  428.21 Acute systolic heart failure  428.22 Chronic systolic heart failure  428.23 Acute on chronic systolic heart failure  428.3 Diastolic heart failure  428.30 Diastolic heart failure, unspecified  428.31 Acute diastolic heart failure  428.32 Chronic diastolic heart failure  428.33 Acute on chronic diastolic heart failure  428.4 Combined systolic and diastolic heart failure  428.40 Combined systolic and diastolic heart failure, unspecified  428.41 Acute combined systolic and diastolic heart failure  428.42 Chronic combined systolic and diastolic heart failure  428.43 Acute on chronic combined systolic and diastolic heart failure  428.9 Heart failure, unspecified | [I50](http://www.icd10data.com/ICD10CM/Codes/I00-I99/I30-I52/I50-/I50) Heart failure  [I50.1](http://www.icd10data.com/ICD10CM/Codes/I00-I99/I30-I52/I50-/I50.1) Left ventricular failure, unspecified  [I50.2](http://www.icd10data.com/ICD10CM/Codes/I00-I99/I30-I52/I50-/I50.2) Systolic (congestive) heart failure  [I50.20](http://www.icd10data.com/ICD10CM/Codes/I00-I99/I30-I52/I50-/I50.20) Unspecified systolic (congestive) heart failure  [I50.21](http://www.icd10data.com/ICD10CM/Codes/I00-I99/I30-I52/I50-/I50.21) Acute systolic (congestive) heart failure  [I50.22](http://www.icd10data.com/ICD10CM/Codes/I00-I99/I30-I52/I50-/I50.22) Chronic systolic (congestive) heart failure  [I50.23](http://www.icd10data.com/ICD10CM/Codes/I00-I99/I30-I52/I50-/I50.23) Acute on chronic systolic (congestive) heart failure  [I50.3](http://www.icd10data.com/ICD10CM/Codes/I00-I99/I30-I52/I50-/I50.3) Diastolic (congestive) heart failure  [I50.30](http://www.icd10data.com/ICD10CM/Codes/I00-I99/I30-I52/I50-/I50.30) Unspecified diastolic (congestive) heart failure  [I50.31](http://www.icd10data.com/ICD10CM/Codes/I00-I99/I30-I52/I50-/I50.31) Acute diastolic (congestive) heart failure  [I50.32](http://www.icd10data.com/ICD10CM/Codes/I00-I99/I30-I52/I50-/I50.32) Chronic diastolic (congestive) heart failure  [I50.33](http://www.icd10data.com/ICD10CM/Codes/I00-I99/I30-I52/I50-/I50.33) Acute on chronic diastolic (congestive) heart failure  [I50.4](http://www.icd10data.com/ICD10CM/Codes/I00-I99/I30-I52/I50-/I50.4) Combined systolic (congestive) and diastolic (congestive) heart failure  [I50.40](http://www.icd10data.com/ICD10CM/Codes/I00-I99/I30-I52/I50-/I50.40) Unspecified combined systolic (congestive) and diastolic (congestive) heart failure  [I50.41](http://www.icd10data.com/ICD10CM/Codes/I00-I99/I30-I52/I50-/I50.41) Acute combined systolic (congestive) and diastolic (congestive) heart failure  [I50.42](http://www.icd10data.com/ICD10CM/Codes/I00-I99/I30-I52/I50-/I50.42) Chronic combined systolic (congestive) and diastolic (congestive) heart failure  [I50.43](http://www.icd10data.com/ICD10CM/Codes/I00-I99/I30-I52/I50-/I50.43) Acute on chronic combined systolic (congestive) and diastolic (congestive) heart failure  [I50.8](http://www.icd10data.com/ICD10CM/Codes/I00-I99/I30-I52/I50-/I50.8) Other heart failure  [I50.81](http://www.icd10data.com/ICD10CM/Codes/I00-I99/I30-I52/I50-/I50.81) Right heart failure  [I50.810](http://www.icd10data.com/ICD10CM/Codes/I00-I99/I30-I52/I50-/I50.810) …… unspecified  [I50.811](http://www.icd10data.com/ICD10CM/Codes/I00-I99/I30-I52/I50-/I50.811) Acute right heart failure  [I50.812](http://www.icd10data.com/ICD10CM/Codes/I00-I99/I30-I52/I50-/I50.812) Chronic right heart failure  [I50.813](http://www.icd10data.com/ICD10CM/Codes/I00-I99/I30-I52/I50-/I50.813) Acute on chronic right heart failure  [I50.814](http://www.icd10data.com/ICD10CM/Codes/I00-I99/I30-I52/I50-/I50.814) …… due to left heart failure  [I50.82](http://www.icd10data.com/ICD10CM/Codes/I00-I99/I30-I52/I50-/I50.82) Biventricular heart failure  [I50.83](http://www.icd10data.com/ICD10CM/Codes/I00-I99/I30-I52/I50-/I50.83) High output heart failure  [I50.84](http://www.icd10data.com/ICD10CM/Codes/I00-I99/I30-I52/I50-/I50.84) End stage heart failure  [I50.89](http://www.icd10data.com/ICD10CM/Codes/I00-I99/I30-I52/I50-/I50.89) Other heart failure  [I50.9](http://www.icd10data.com/ICD10CM/Codes/I00-I99/I30-I52/I50-/I50.9) Heart failure, unspecified |
| Myocarditis | 422.0 Acute myocarditis in diseases classified elsewhere | I51.4, Myocarditis, unspecified  [I40](https://www.icd10data.com/ICD10CM/Codes/I00-I99/I30-I52/I40-/I40) Acute myocarditis  [I40.1](https://www.icd10data.com/ICD10CM/Codes/I00-I99/I30-I52/I40-/I40.1) Isolated myocarditis  [I40.8](https://www.icd10data.com/ICD10CM/Codes/I00-I99/I30-I52/I40-/I40.8) Other acute myocarditis  [I40.9](https://www.icd10data.com/ICD10CM/Codes/I00-I99/I30-I52/I40-/I40.9) Acute myocarditis, unspecified |
| Arrhythmia | 427 Cardiac dysrhythmias  427.0 Paroxysmal supraventricular tachycardia  427.1 Paroxysmal ventricular tachycardia  427.2 Paroxysmal tachycardia, unspecified  427.3 Atrial fibrillation and flutter  427.31 Atrial fibrillation  427.32 Atrial flutter  427.4 Ventricular fibrillation and flutter  427.41 Ventricular fibrillation  427.42 Ventricular flutter  427.5 Cardiac arrest  427.6 Premature beats  427.60 Premature beats, unspecified  427.61 Supraventricular premature beats  427.69 Other premature beats  427.8 Other specified cardiac dysrhythmias  427.81 Sinoatrial node dysfunction  427.89 Other specified cardiac dysrhythmias  427.9 Cardiac dysrhythmia, unspecified | I47 Paroxysmal tachycardia  I47.0 Re-entry ventricular arrhythmia  I47.1 Supraventricular tachycardia  I47.2 Ventricular tachycardia  I47.9 Paroxysmal tachycardia, unspecified  I48 Atrial fibrillation and flutter  I48.0 Paroxysmal atrial fibrillation  I48.1 Persistent atrial fibrillation  I48.2 Chronic atrial fibrillation  I48.3 Typical atrial flutter  I48.4 Atypical atrial flutter  I48.9 Unspecified atrial fibrillation and atrial flutter  I48.91 Unspecified atrial fibrillation  I48.92 Unspecified atrial flutter  I49 Other cardiac arrhythmias  I49.0 Ventricular fibrillation and flutter  I49.01 Ventricular fibrillation  I49.02 Ventricular flutter  I49.1 Atrial premature depolarization  I49.2 Junctional premature depolarization  I49.3 Ventricular premature depolarization  I49.4 Other and unspecified premature depolarization  I49.40 Unspecified premature depolarization  I49.49 Other premature depolarization  I49.5 Sick sinus syndrome  I49.8 Other specified cardiac arrhythmias  I49.9 Cardiac arrhythmia, unspecified |
| Pericardial disease | 420 Acute pericarditis  420.0 Acute pericarditis in diseases classified elsewhere  420.9 Other and unspecified acute pericarditis  420.90 Acute pericarditis, unspecified  420.91 Acute idiopathic pericarditis  420.99 Other acute pericarditis  423 Other diseases of pericardium  423.0 Hemopericardium  423.1 Adhesive pericarditis  423.2 Constrictive pericarditis  423.3 Cardiac tamponade  423.8 Other specified diseases of pericardium  423.9 Unspecified disease of pericardium | I30 Acute pericarditis  I30.0 Acute nonspecific idiopathic pericarditis  I30.8 Other forms of acute pericarditis  I30.9 Acute pericarditis, unspecified  I31 Other diseases of pericardium  I31.0 Chronic adhesive pericarditis  I31.1 Chronic constrictive pericarditis  I31.2 Hemopericardium, not elsewhere classified  I31.3 Pericardial effusion (noninflammatory)  I31.4 Cardiac tamponade  I31.8 Other specified diseases of pericardium  I31.9 Disease of pericardium, unspecified |
| Heart Block | 426 Conduction disorders  426.0 Atrioventricular block, complete  426.1 Atrioventricular block other and unspecified  426.10 Atrioventricular block, unspecified  426.11 First degree atrioventricular block  426.12 Mobitz (type) II atrioventricular block  426.13 Other second degree atrioventricular block  426.2 Left bundle branch hemiblock  426.3 Other left bundle branch block  426.4 Right bundle branch block  426.5 Bundle branch block other and unspecified  426.50 Bundle branch block, unspecified  426.51 Right bundle branch block and left posterior fascicular block  426.52 Right bundle branch block and left anterior fascicular block  426.53 Other bilateral bundle branch block  426.54 Trifascicular block  426.6 Other heart block  426.7 Anomalous atrioventricular excitation  426.8 Other specified conduction disorders 426.89 Other specified conduction disorders  426.9 Conduction disorder, unspecified | I44 Atrioventricular and left bundle-branch block  I44.0 Atrioventricular block, first degree  I44.1 Atrioventricular block, second degree  I44.2 Atrioventricular block, complete  I44.3 Other and unspecified atrioventricular block  I44.30 Unspecified atrioventricular block  I44.39 Other atrioventricular block  I44.4 Left anterior fascicular block  I44.5 Left posterior fascicular block  I44.6 Other and unspecified fascicular block  I44.60 Unspecified fascicular block  I44.69 Other fascicular block  I44.7 Left bundle-branch block, unspecified  I45 Other conduction disorders  I45.0 Right fascicular block  I45.1 Other and unspecified right bundle-branch block  I45.10 Unspecified right bundle-branch block  I45.19 Other right bundle-branch block  I45.2 Bifascicular block  I45.3 Trifascicular block  I45.4 Nonspecific intraventricular block  I45.5 Other specified heart block  I45.81 Long QT syndrome  I45.89 Other specified conduction disorders  I45.9 Conduction disorder, unspecified |
